# Insights into the Regulation of Algal Proteins and Bioactive Peptides Using Proteomic and Transcriptomic Approaches

**DOI:** 10.3390/molecules24091708

**Published:** 2019-05-02

**Authors:** Lucie Beaulieu

**Affiliations:** Département des Sciences des Aliments, Institut sur la Nutrition et les Aliments Fonctionnels (INAF), 2425, rue de l’Agriculture, Université Laval, Québec, QC G1V 0A6, Canada; lucie.beaulieu@fsaa.ulaval.ca; Tel.: +1-418-656-2131 (#404767)

**Keywords:** edible macroalgae, protein quality, bioactive peptides, metabolic pathways, proteomics, transcriptomics, health food

## Abstract

Oceans abound in resources of various kinds for R&D and for commercial applications. Monitoring and bioprospecting allow the identification of an increasing number of key natural resources. Macroalgae are essential elements of marine ecosystems as well as a natural resource influenced by dynamic environmental factors. They are not only nutritionally attractive but have also demonstrated potential health benefits such as antioxidant, antihypertensive, and anti-inflammatory activities. Several bioactive peptides have been observed following enzymatic hydrolysis of macroalgal proteins. In addition, significant differences in protein bioactivities and peptide extracts of wild and cultivated macroalgae have been highlighted, but the metabolic pathways giving rise to these bioactive molecules remain largely elusive. Surprisingly, the biochemistry that underlies the environmental stress tolerance of macroalgae has not been well investigated and remains poorly understood. Proteomic and functional genomic approaches based on identifying precursor proteins and bioactive peptides of macroalgae through integrated multi-omics analysis can give insights into their regulation as influenced by abiotic factors. These strategies allow evaluating the proteomics profile of regulation of macroalgae in response to different growth conditions as well as establishing a comparative transcriptome profiling targeting structural protein-coding genes. Elucidation of biochemical pathways in macroalgae could provide an innovative means of enhancing the protein quality of edible macroalgae. This could be ultimately viewed as a powerful way to drive the development of a tailored production and extraction of high value molecules. This review provides an overview of algal proteins and bioactive peptide characterization using proteomics and transcriptomic analyses.

## 1. Introduction

Macroalgae (i.e., seaweeds) are essential elements of marine ecosystems delivering a range of ecologically and economically valuable biological services. In addition to their fundamental ecological roles in providing habitat for fish and invertebrates, they play an economic role through their use as food, either as sources of basic ingredients or as dietary supplements, with an estimated economic value of US $6.4 billion [1]. Macroalgae are well known for their abundance in fibers, minerals (e.g., iodine), and certain vitamins (e.g., B12) but also contain numerous proteins/peptides, polyphenols, and polyunsaturated fatty acids (PUFAs) [2]. As a source of high-value-added compounds, macroalgae are of great interest to the scientific community, to the food industry, and to consumers. They are exploited for numerous applications in the food, cosmetics, agricultural and horticultural sectors, as well as in bioenergy and human health [3]. Marine macroalgae, which have evolved independently from higher plants, produce numerous, unique metabolites that are not found in terrestrial plants [4]. The chemical composition of macroalgae can also be quite variable according to environmental parameters such as light, temperature, drought, nutrients, and salinity [5]. They are also exposed to anthropogenic stressors associated with climate change, invasive species, and pathogens [6]. Rapid changes in the pools of structural proteins and enzymes occur as adaptive physiological responses of macroalgae to their environment. These abiotic factors have contributed to driving the regulation of biochemical pathways in macroalgae [4,7] and to the production of biologically active secondary metabolites [8,9]. The biological functions of many algal compounds are, however, only partially understood, and research on the chemical diversity and ecology of algae is limited, although studies assessing their commercial value are growing rapidly [3].

As bioactivities from macroalgae are associated with specific compounds, it is crucial to identify the chemical diversity of this resource, quantify the compounds of interest, and monitor their variability as a function of environmental and potentially anthropogenic stresses that induce measurable physiological changes [10]. Hence, in response to their environment, macroalgae produce biomarkers such as polyphenols [11], antioxidants [12], PUFAs of the omega-3 type [13], and heat shock proteins [14,15,16], which are, among other things, indicators of quality of the macroalgae. Studies on proteinaceous compounds as biomarkers, in response to environmental factors, have covered several macroalgae species from tropical [17] to arctic environments through temperate zones [18]. In the North, a few reports have come from samples in the Bay of Fundy [19], and from the north Gulf of Saint Lawrence exposed to extreme climatic conditions [20]. This algal resource has been recently promoted for its nutritional protein quality, which is mainly defined by its amino acid composition, digestibility, and health benefit properties [21,22]. Recent results suggest that cultivation of macroalgae species using selected conditions of photoperiod, temperature, and nutrient content is a promising strategy to control algal physiology and, therefore, to enhance the production/activity of targeted bioactive proteinaceous compounds [19,20]. Tissue-wide changes in cellular structure, function, and activity can be inferred through proteomic analysis, but only a limited number of studies have investigated how proteomic regulation in macroalgae can be influenced by environmental stresses [23,24,25,26,27]. Better knowledge of both quality and traceability of algal-derived proteins could promote the growth conditions, allowing the production of high levels of bioactive proteins and peptides, leading to cultivation improvement. Indeed, proteomics are increasingly being utilized to profile expressed proteins in different foods to address their safety [28]. In this context, controlled acclimatization studies are useful for unravelling the regulation of various proteins in macroalgae using a proteomics and functional genomics approach, giving insights into how these organisms cope with environmental stresses.

**Figure 1:**
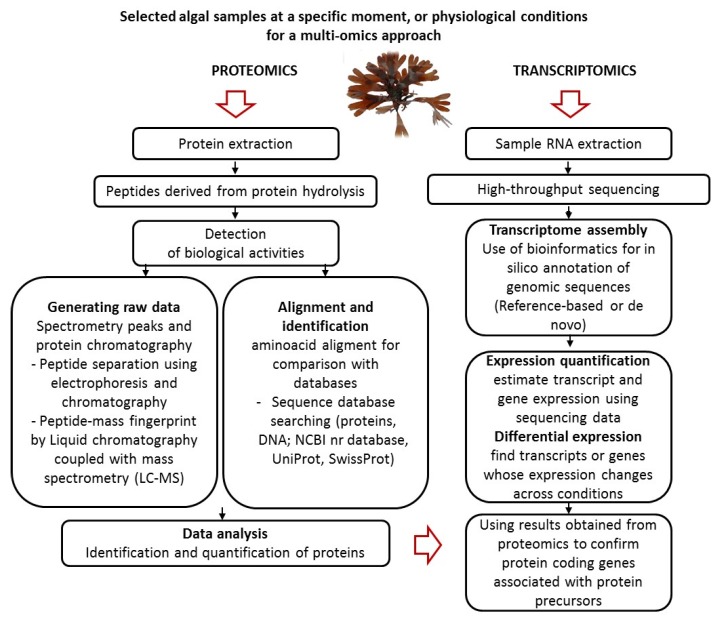
General strategy for the characterization of macroalgal proteins and bioactive peptides and the determination of their gene coding sequence using proteomic and transcriptomic analyses.

Thus, advancements in DNA and RNA sequencing along with mass spectrometry (MS), supported by high-performance computing, allowed the identification of new metabolic processes in many organisms, expanding the knowledge of biological systems [29]. Large-scale biological data together with associated bioinformatics analysis are known by the “-omics” suffix and comprise genomics, metagenomics, phylogenomics, transcriptomics, proteomics, and metabolomics [29]. In the present review, the application of proteomic and transcriptomic fields in macroalgae research will be particularly discussed. As represented in Figure 1, in order to illustrate the application of these -omics technologies, macroalgae have been chosen to demonstrate how their proteins and bioactive peptides may be monitored, by analyzing biological samples at a specific moment, or physiological condition of interest. This strategy will ultimately support optimization of their production.

## 2. Macroalgal Proteins and their Bioactive Derived Peptides

Due to the increasing human population, food security remains a major concern [28]. The search for unconventional protein sources such as algae has been more and more investigated [22,30,31]. Proteins are present in macroalgae in various cellular compartments as enzymes or as intracellular components or can be bound to pigments and polysaccharides [3]. Macroalgae have relatively high levels of proteins (up to 47% dry weight), and the content depends on the species, season, and nutrient supply during the growth phase [4,20,31,32,33,34,35]. The algal proteins that have been studied the most belong to the class of phycobiliproteins (phycoerythrin, phycocyanins, and allophycocyanins) and lectins, two main known groups of functionally active proteins [3,36,37,38]. Phycobiliproteins are a family of relatively stable and greatly soluble fluorescent proteins present in red algae. These proteins play a biological role in collecting light, and through fluorescence resonance energy transfer, which is implied in the photosynthesis [38]. Phycobiliproteins are used as dyes in food, cosmetics, and as fluorescent markers in biomedical research [3]. The lectins have been isolated from red and green macroalgae [38] and exhibit affinity for carbohydrates and glycoproteins as well as participate in many biological processes like intercellular communication. The lectins have the capacity to inhibit the growth of marine *Vibrio* strains [39] and also possess potential antiviral, anti-inflammatory, anticancer, and anti-HIV activities [36,40].

Algal protein composition, structure, and potential bioactivities remain conversely much less characterized than other algal components such as polysaccharides, phenols, and pigments [41]. Several bioactive peptides have been obtained and characterized following enzymatic hydrolysis of food proteins, but so far, only limited research efforts have focused on edible macroalgal proteins [41]. Numerous recent studies have shown that enzymatic hydrolysis of macroalgal extracts generates a new range of peptides with various biological activities [42]. This general strategy includes proteolysis of proteins, activity tests, identification of peptide sequences by in silico analyses using databases (NCBI nr (non-redundant), UniProt, SwissProt), and confirmation of the activity using synthetic peptides [43,44,45]. In order to confirm the presence of potential bioactive peptides in these extracts, a comparison of the identified peptide sequences with known bioactive peptides in online databases could be done (e.g., Biopep-uwm, APD and CAMP) [44,45]. Some of those typical studies are listed in Table 1.

For instance, peptidic inhibitors of the angiotensin-converting enzyme (ACE), an enzyme involved in blood pressure regulation, derived from protein hydrolysates, have been found in *Porphyra* [49,50,51,52], *Palmaria* [20,43,45], *Undaria*, and *Sargassum* species [57,58,59]. Antioxidant peptides have been isolated from *Palmaria* [20,33,43,46,47], *Porphyra* [49,53,54], *Undaria* [60], and *Ecklonia* species [61]. Bioassay-guided approaches combined with MS analysis and database searches have allowed identification of bioactive peptide sequences from *Palmaria palmata* [43,47]. Those have been associated with their precursor proteins, which are notably the enzyme ribulose-1,5-diphosphate carboxylase/oxygenase (RubisCo), a plant enzyme involved in photosynthesis, and pigments belonging to the class of phycobiliproteins (allophycocyanin, phycocyanin, phycoerythrin). Other bioactive peptides have been identified in algal protein hydrolysates, such as both antihypertensive renin inhibitory peptides [48,62], correlated to both photosystem I and II proteins, and dipeptidyl peptidase (DPP) IV inhibitory peptides [63]. Beaulieu et al. (2015) [44] extracted antibacterial peptides from the brown macroalgae *Saccharina longicruris*, and the identified peptides, by tandem mass spectrometry (MS/MS) and sequence database searching, have been associated with precursor proteins similar to ubiquitin, leucine rich repeat protein, histone, and a ribosomal structure, which form part of the innate immune defense of the macroalgae.

A study demonstrated that the health value of bread was increased through the addition of a macroalgae renin inhibitory *P. palmata* protein hydrolysate [64], showing that bioactive peptides from macroalgae are valuable components which could have applications in food products. Bioactive peptides may also be released by digestive enzymes. For this reason, it is necessary to generate information on absolute amounts of these peptides to establish the basis of proven bioavailability of a given ingredient in the original food matrix as well as in the relevant body fluids or tissues [28].

Therefore, in spite of being a great potential source of active peptides, protein hydrolysates from macroalgae in general have received limited attention globally. Recent experiments have shown that culture conditions, namely the addition of nutrients (NH_4_^+^ as a source of nitrogen and PO_4_^−^ as a source of phosphorus), would yield hydrolysates with a greater biological activity [20]. These findings highlight the potential of using proteomic approaches for the investigation of algal proteins and derived peptides targeting selected proteinaceous biomarkers as quality indicators.

## 3. Proteomic Profile Analysis of Macroalgae

Much research has identified and characterized sets of proteins composing the proteome of a cell, an organelle or a tissue [29]. As mentioned previously, prior to MS analysis, proteins extracted from a biological sample may be separated and digested followed by peptide sequencing using MS/MS and automated database searching. Depending on the means of protein isoelectric point and molecular weight, two-dimensional (2-D) gel electrophoresis can be used for protein separation [65]. Complementary separation steps such as liquid chromatography (LC) techniques could be used to reduce the complexity of a peptide mixture based on the physicochemical properties of peptides such as mass, charge, and hydrophobicity [66]. Information about proteomics methods can be found in detail in a review about the application of the “-omics” fields in biomass research [29].

Functional integration of “-omics” disciplines and systems biology approaches have been applied to the study of terrestrial plants, but scarcely for marine macrophytes [6], and proteomics studies in the macroalgae research fields are still in their infancy. Global proteome analysis of plants by means of peptide libraries has been reviewed by Righetti and Boschetti (2016) [67]. In cases where an important number of polysaccharides is present in plants such as in macroalgae, the proteins are present particularly at low concentrations. Consequently, the functional investigation of protein differential biosynthesis in response to various environmental stresses is really challenging [67]. Among the limited reports on macroalgae proteome to date, the proteome of *Gracilaria* *changii*, a red alga of economic importance, used in poke, a raw fish salad served as an appetizer in Hawaiian cuisine, has been described using 2-D gel electrophoresis and MS analysis [68]. A few key proteins have been identified, including pigment proteins (phycobiliproteins), metabolic enzymes, and ion transporters. For *Scytosiphon gracilis* and *Ectocarpus siliculosus*, proteins identified by similar strategies are reported to be involved in primary metabolic pathways, including carbon fixation, protein synthesis, and oxidative phosphorylation [69]. Table 2 shows some examples of proteomic approaches used with investigation of how protein regulation in macroalgae can be influenced by environmental stresses. A bottom–up approach, where smaller peptides derived from enzymatic digestion (trypsin) of proteins are generally used followed by a pre-fractionation in 2-D gel electrophoresis, LC-MS analysis (mostly Matrix Assisted Laser Desorption Ionisation–Time of Flight: MALDI-TOF MS) and amino acid alignment and identification for comparison with several databases (e.g., NCBI, UniProt, SwissProt). One of these works has studied the proteome responses of a red alga, *Gracilaria lemaneiformis*, exposed to lead stress [70]. A number of fourteen Pb stress-regulated proteins were identified and are known to be involved in different cellular functions, such as photosynthesis, energy and protein metabolism, carbohydrate transport and metabolism, signal transduction, as well as antioxidation [70]. This proteomic approach has provided a better picture of protein networks and metabolic pathways involved in defense mechanisms. Another study has indicated that cadmium stress negatively affects the metabolic activity of *Sargassum fusiforme* through the downregulation of key metabolic enzymes such as RubisCo [71]. The identification of proteins implicated in desiccation tolerance in the red alga *Pyropia orbicularis* and in high-temperature stress in *Pyropia haitanensuis* have also been possible by a similar proteomic approach [23]. However, only a limited number of studies have investigated how proteomic regulation in macroalgae can be influenced by physical, chemical, and environmental stresses [23,24,25,26,27]. From those studies, it is clear, however, that seasonal impact, pH, high temperature, and excess minerals all induce proteomic changes. According to this kind of comparative proteomic study, it is possible to identify candidate stress-responsive proteins and enzymatic pathways contributing significantly to the understanding of the molecular mechanisms underlying tolerance to stress. In addition, the determination of protein/peptides mass fingerprints is of relevance in terms of ultimately being able to link precursor protein expression with the ultimate release of bioactive peptides. These studies could help to increase knowledge on macroalgae sustainability and provide the baseline and tools for establishing their potential applications. Finding the peptide sequences in the protein sequences from genomes, for example, could unveil functional peptides from natural sources, as it has been recently demonstrated by Kumagai et al. 2019 [72]. In this study, the complete plastid genome sequence of *Palmaria* sp. (Japan) and annotated protein-coding genes have been determined. In silico analysis has allowed studying the relationship between proteins from the plastid genome of this alga and ACE inhibitory peptides. Thus, the combined use of proteomics and genomic approaches, such as transcriptomics, constitutes a challenging approach for the investigation of algal proteins and for further identification and quantification of gene expression and gene products related to proteins and bioactive peptides in macroalgae. Effectively, integration of proteomics with other technologies, such as RNA-seq, has helped the identification of genes and transcripts that appear to encode proteins, which had a lack of direct experimental evidence [73]. For instance, an RNA-seq approach associated with mass spectrometry was successfully conducted to assess the primary sequence of active hydrolytic peptides issued from hydrolysates of white shrimp (*Litopenaeus vannamei*) byproducts [74].

## 4. Transcriptomic Analysis of Macroalgae in Response to Abiotic Factors

RNA molecules have fundamental roles as intermediates between the genomic information and the proteome (messenger RNAs, or mRNAs) [29]. The transcriptome consists of a set of expressed RNA molecules, which can be studied using high-throughput RNA-sequencing technologies (RNA-Seq), microarrays, and expressed sequence tags (EST), among others [75]. RNA-Seq data are widely used to discover and classify transcripts, to annotate a gene structure, and to quantify the transcript abundance change when comparing different biological samples at a specific moment or target conditions [29]. Information about transcriptomic analysis can be found in detail in another review [75].

A system approach that combines both gene expression and production of metabolites associated with these genes has the potential to uncover the regulation of biochemical pathways of algae not revealed by entire genome sequencing [76]. Only a few studies in integrative analysis have been carried out on the various adaptive physiological responses of macroalgae. Table 3 shows several transcriptomic approaches according to the investigation of how gene expression in macroalgae can be influenced by abiotic stress. After preparing the experiment to answer the question of interest, the transcriptome assembly can be achieved through a reference-based (map reads to the genome transcripts assembly) or de novo (without a genome) method. Numerous databases have been used by different authors as shown in Table 3. Then, estimate transcript and gene expression using sequencing data allow the expression quantification and support finding transcripts or genes whose expression changes across conditions.

For instance, RNA-Seq was used to profile transcriptomic changes in *Saccharina japonica* under different Cu^2+^ concentrations [77]. The suppression of diverse essential biological processes such as photosynthesis, protein synthesis, redox activity, and metabolism and biosynthesis of functional biomolecules have been observed at the transcriptional level, which could be associated with the toxicity of Cu^2+^ in this alga. In addition, effects of UV radiation at different temperatures have been assessed to study the influence of growth conditions on the acclimation to stress of the brown macroalga *Saccharina latissima* on the transcriptional level [80]. These results have suggested that low temperatures caused metabolic alterations to increase stress performance of *S*. *latissima*. A variable metabolic response of another brown macroalga *Sargassum vulgare* at different time scales to natural acidification has also been observed, and changes in the levels of sugars, fatty acids, amino acids, antioxidants, and phenolic compounds have been examined [81]. In addition, a parallel analysis of proteins has been conducted in the brown macroalga *Sargassum fusiforme* responding to hyposalinity stress using a comparative physiology approach combining methods of systems biology, including proteomics [82]. These results revealed 51 differentially expressed protein spots in *S. fusiforme*, most of which were enzymes involved in photosynthesis, carbohydrate metabolism, and energy metabolism. The response of a red macroalgae *Gracilaria lemaneiformis* to nitrogen (N) deprivation was studied and proteomics data indicated an important increase of electron-transfer proteins in the photosynthesis, but phycoerythrin protein decreased under N deprivation [83]. However, glutamine synthetase protein and RubisCO protein remained stable. The transcriptome changes that occur during acclimation of brown algae of the genus *Ectocarpus* were investigated with three different abiotic stresses (hyposaline, hypersaline, and oxidative stress) [79]. The effects of these changes in the concentrations of various metabolites (urea, amino acids, sugars, polyols) were also studied and correlated very well with transcriptomic data [84]. The sequencing of the *Ectocarpus siliculosus* genome [85] enabled such novel approaches to study the mechanisms involved in stress tolerance. It was also shown that transcriptomic analyses can be easily performed based on the reference genome of a related species. For example, the assembly of all sequences derived from the red macroalga *Laurencia dendroidea* was aligned against the *Florideophyceae* EST NCBI database [78]. The complete mitochondrial genome of some red macroalgae was sequenced [86], including that of *P. palmata* with 29,735 bp in length (NCBI Reference Sequence: NC_026056.1), but the whole genome of *P. palmata* has yet to be annotated. In addition, sequencing of the 105-Mbp genome of the red macroalga *Chondrus crispus* and the annotation of some 9606 genes have been reported [87]. Recently, the first whole-genome sequence of a green macroalga, *Ulva mutabilis*, has also been reported [88].

Transcriptomic analysis allows understanding how macroalgae adapts to abiotic stresses, and it could promote the identification of numerous stress-related genes and proteins and the pathways in which they function. This strategy aims to link these metabolic pathways to precursor proteins and their bioactive peptides. Correlations between RNA levels and protein levels are sometimes non-meaningful, but it has been demonstrated in algae, by some authors, that most of several metabolic changes in response to stress corresponded well to observations made in the transcriptome [84]. These findings pave the way for further studies of the biosynthesis of proteinaceous biomarkers and of the role of these potential signaling molecules, including their implication in the abiotic stress response in macroalgae [89]. All these new developments give the opportunity to define the metabolic pathways for bioactive peptides in macroalgae, which can be deciphered concurrently at the genomic, proteomics, and physiological levels. Thus, the evidence obtained on the expression of genes involved in bioactive peptide synthesis could be correlated to their metabolic pathways in order to optimize the biosynthesis of active alga-based foods according to abiotic factors.

## 5. Conclusions and Perspectives

The complexity of biological systems is a factor to consider for performing an integrated multi-omics analysis using data such as gene expression data (transcriptomics) and protein quantification (proteomics). The outcomes of proteomics and functional genomic approaches can be of major relevance to leading the valorization of macroalgae. Basic information could be generated through these strategies aimed at deciphering the environmental conditions leading to the production of high-quality proteins and bioactive peptides in macroalgae. The generated knowledge could provide essential information to the understanding of how the environment affects the physiological adaptive response and metabolism of macroalgae; to the expression of genes involved in the synthesis of quality proteins, and on the structure–activity relationships of bioactive peptides by elucidating their amino acid sequences, which supports further bioinformatics studies to molecular modelling of peptides. Consolidating a system-wide approach using -omics technologies could help to develop a greater understanding of how bioactive proteins and peptides originated from macroalgae may ultimately be optimized by carefully manipulating the environmental conditions. This approach can be viewed as a powerful way to discover the potential of edible macroalgal resources to drive the development of oriented cultivation based on the tailored production and extraction of high value molecules. Thus, the successful outcomes of these studies can be of significant impact to the algae industry sector.

## Figures and Tables

**Table 1:** Bioactivity of peptides derived from marine macroalgae protein hydrolysates.

Macroalgae	Activity	References
*Palmaria palmata* (red algae)	Cardioprotective, antidiabetic, antioxidant	[46]
	Angiotensin converting enzyme (ACE) inhibitor, antioxidant	[20,33,43,45]
	Antioxidant	[47]
	Renin inhibitor	[48]
*Porphyra columbina* (red algae)	Antioxydant, ACE inhibitor	[49,50]
*Porphyra* (*Pyropia*) *yezoensis* (red algae)	ACE inhibitor	[51,52]
*Porphyra* (*Pyropia*) *tenera* (red algae)	Antioxidant, anti-acetylcholinesterase, anti-inflammatory	[53]
*Porphyra dioica* (red algae)	Anti-hypertensive, antidiabetic, antioxidant	[54]
*Porphyra haitanesis* (red algae)	Anti-proliferative	[55,56]
*Sargassum fusiformis* (brown algae)	ACE inhibitor	[57]
*Undaria pinnatifida* (brown algae)	ACE inhibitor	[58,59]
	Antioxidant	[60]
*Ecklonia cava* (brown algae)	Antioxidant	[61]
*Saccharina longicruris* (brown algae)	Antibacterial	[44]

**Table 2:** Typical Proteomic approaches according to the investigation of how protein regulation in macroalgae can be influenced by abiotic stress.

Macroalgae	Proteomic Approaches	Generating Raw Data	Alignment and Identification	Data Analysis	Reference
*Gracilaria lemaneiformis (red algae)*	Proteins analyzed by MS by a bottom–up approach (smaller peptides derived from enzymatic digestion of proteins)	2D-Electrophoresis; MALDI-TOF MS	Mascot aligner; MoverZ and NCBI non-redundant protein database	Protein identification was accepted with a MASCOT score higher than 60 with more than five matched peptides. The MASCOT protein search was performed via all plants’ database.	[70]
*Pyropia haitanensis *(red algae)	Proteins analyzed by MS by a bottom–up approach	2D-Electrophoresis; MALDI-TOF/TOF MS	Mascot aligner; NCBI and SwissProt database	According to the search engine parameters, scores greater than 65 (*p* < 0.05) were considered positive.	[25]
*Pyropia orbicularis *(red algae)	Proteins analyzed by MS by a bottom–up approach	2D-Electrophoresis; Nano-LC-MS/MS coupled on-line to a LTQ Orbitrap Discovery system mass spectrometer	PEAKS Studio software. This database included *P. orbicularis* ESTs, *Chondrus crispus *genes, and *Ectocarpus siliculosus* genes, BLAST-P in NCBI database	ExPASy Compute pI/MW tool; Protein functional classification using KEGG pathway analysis. The threshold was set to a minimal Significance of 1 × 10^−3^ and an identity percentage of greater than 25%.	[23]
*Sargassum fusiforme *(brown algae)	Proteins analyzed by MS by a bottom–up approach	2D-Electrophoresis; MALDI-TOF/TOF MS	Mascot aligner; NCBI non-redundant FASTA database and UniProt database	Protein functional classification using KEGG pathway analysis; Protein-protein association information evaluated with the STRING database against *Phaeodactylum tricornutum* database. Individual ion scores of more than 28 indicate identity or extensive homology (*p* < 0.05).	[71]

**Table 3:** Typical transcriptomic approaches according to the investigation of how gene expression in macroalgae can be influenced by abiotic stress.

Macroalgae	Planning and Data Generation	Transcriptome Assembly	Expression Quantification	Differential Expression	Reference
*Saccharina japonica* (brown algae)	Copper treatments were conducted by transferring the juvenile sporophytes to fresh seawater with final Cu^2+^ concentrations of 10, 100, and 200 μg L^−1^. Illumina Hiseq sequencing.	De novo transcriptome assembly with Trinity. Functional annotation using the basic local alignment search tool and a translated nucleotide query (BLASTX) against the non-redundant protein and non-redundant nucleotide databases of the NCBI, Protein family, SwissProt, eukaryotic Ortholog Groups (KOG), and the KEGG databases. Functional annotation by Gene Ontology (GO) was performed using Blast2GO software.	Transcript quantification with RSEM. Validation of the differentially expressed genes (DEGs) by RT-qPCR.	Compared with the control, the number of DEGs was 11,350 (4944 up- and 6406 downregulated) in the 200 μg L^−1^ treatment group and 2868 (1075 up- and 1793 downregulated) in the 100 μg L^−1^ treatment group, whereas much fewer DEGs were detected in the 10 μg L^−1^ treatment group.	[77]
*Laurencia dendroidea* (red algae)	Three specimens of *L. dendroidea* collected in the intertidal zone during high tide. The EST sequences deposited for the class. Florideophyceae in the NCBI were downloaded and the reads were assembled using the TGICL software from TIGR.	The assembly was aligned against the Florideophyceae. EST NCBI database. Taxonomic and functional analysis performed on assembled sequences using the Newbler software, and annotated, using the MG-RAST server, through BLAST, against the GenBank, COG, KEGG and Subsystems databases.	PCR amplification.	A total of 6 transcriptomes were obtained from specimens of *L. dendroidea* sampled in three different coastal locations of Rio de Janeiro state.	[78]
*Ectocarpus siliculosus* (brown algae)	Three different stresses: (hyposaline, hypersaline, oxidative). 90,637 EST sequences used for the microarray design.	Sequences were annotated with KEGG orthology (KO) numbers using KOBAS and with GO terms using GOPET. Protein sequences corresponding to the assembled EST sequences were predicted using ORF predictor.	RT-qPCR validation of the microarray.	70% of the expressed genes are regulated in response to at least one of these stressors.	[79]
